# Observations on Abundance of Bluntnose Sixgill Sharks, *Hexanchus griseus*, in an Urban Waterway in Puget Sound, 2003-2005

**DOI:** 10.1371/journal.pone.0087081

**Published:** 2014-01-27

**Authors:** Denise Griffing, Shawn Larson, Joel Hollander, Tim Carpenter, Jeff Christiansen, Charles Doss

**Affiliations:** 1 Life Sciences, Seattle Aquarium, Seattle, Washington, United States of America; 2 Department of Statistics, University of Washington, Seattle, Washington, United States of America; University of California Davis, United States of America

## Abstract

The bluntnose sixgill shark, *Hexanchus griseus*, is a widely distributed but poorly understood large, apex predator. Anecdotal reports of diver-shark encounters in the late 1990’s and early 2000’s in the Pacific Northwest stimulated interest in the normally deep-dwelling shark and its presence in the shallow waters of Puget Sound. Analysis of underwater video documenting sharks at the Seattle Aquarium’s sixgill research site in Elliott Bay and mark-resight techniques were used to answer research questions about abundance and seasonality. Seasonal changes in relative abundance in Puget Sound from 2003–2005 are reported here. At the Seattle Aquarium study site, 45 sixgills were tagged with modified Floy visual marker tags, along with an estimated 197 observations of untagged sharks plus 31 returning tagged sharks, for a total of 273 sixgill observations recorded. A mark-resight statistical model based on analysis of underwater video estimated a range of abundance from a high of 98 sharks seen in July of 2004 to a low of 32 sharks seen in March of 2004. Both analyses found sixgills significantly more abundant in the summer months at the Seattle Aquarium’s research station.

## Introduction

Shark populations are in decline worldwide due to overharvest from shark finning, by-catch, entanglement, habitat loss and environmental degradation [1], [2]. Many large sharks are wide ranging occurring in most of the world’s oceans such as the broadnose sevengill (*Notorynchus cepedianus*), spiny dogfish (*Squalus acanthias*), the great white shark (*Carcharodon carcharias*), the blue shark (*Prionace glauca*), and the bluntnose sixgill shark (*Hexanchus griseus*) [1], [3], [4]. Yet in spite of the widespread distribution all of these sharks, all are species at risk because of life histories that include late maturity, low reproductive capacity and their potential vulnerability to overharvest. The population status and the impact of fisheries on these sharks remains unknown prompting their listing as either data deficient (broadnose sevengill), vulnerable (great white shark and spiny dogfish), or near threatened (blue shark and sixgill) by the International Union for the Conservation of Nature (IUCN) [5].

The bluntnose sixgill is found in tropical and temperate waters ranging from shallow coastal waters to the continental slopes and down to abyssal depths [4]. Life history characteristics include slow growth, late reproductive maturity at approximately 20 years and unknown longevity [1], [4], [6], [7]. Distinguishing physical characteristics include six gill slits, a single dorsal fin located posteriorly on the body, and a sub-terminal mouth with dimorphic tooth patterns in the upper and lower jaws [4], [6]. Females are ovoviviparous bearing between 22–108 pups with unknown gestation and reproductive frequency [6]. Newborn sixgill pups are typically 0.7 m while adults may reach a maximum length of 6 m, with females larger than males [1], [8]. Subadult sixgills are defined as males less than 3 m and females less than 4 m [6], [9].

The sixgills’ depth range is from the surface waters to 3000 m [1], [4], [6]. Although they are thought to be primarily bottom and deep dwelling, they have been reported occurring in shallow estuaries in the United States such as Puget Sound in Washington [7], [10] and San Francisco Bay in California [1], [8] as well as the Georgia Basin in British Columbia, Canada [7]. The sixgills’ presence in the shallow waters of Puget Sound in the late 1990’s through 2000 led to an increase in anecdotal reports of encounters between sixgill sharks and divers. These divers noted they were more likely to encounter sixgills in summer months than in winter. It was unknown whether this indicated a true seasonal change in sixgill behavior or was an artifact of changing diver effort with many divers only diving in the summer. In addition, in the summer of 2000, directed fishing for sharks by local recreational fishers resulted in the catch of several sixgills from an area in Elliott Bay near Alki Point where divers had reported frequent encounters. Underwater video and still photography revealed that numerous sightings were made of the same individual sharks at that location over several months. After this directed fishing activity, diver sightings of sixgills in that area ceased for at least three months, sparking questions about the abundance of sixgills in Puget Sound.

This event also stimulated the Seattle Aquarium's (SA) interest to study this normally deep-dwelling shark using simple minimally invasive techniques, such as SCUBA and underwater video cameras, without incurring the expenses normally associated with deep ocean research such as using a submersible. Prior to this event, there were no catch limits for the sixgills in Washington waters. However due to the public’s, as well as the SA’s, concern over the potential overexploitation of local sixgills, Washington Department of Fish and Wildlife (WDFW) regulators responded by placing a temporary closure (later made permanent) on the taking of sixgills in Puget Sound. State fisheries biologists pointed to the lack of information on abundance, movement patterns, and biological parameters of sixgill sharks in Puget Sound dictating a cautious approach to their harvest.

To gather basic information about sixgill sharks in Washington waters, specifically the inland waters of Puget Sound, a joint research team was established in 2002. This team included representatives of National Oceanic and Atmospheric Administration’s (NOAA) National Marine Fisheries Service (NMFS), WDFW, University of Washington (UW), SA, and Point Defiance Zoo and Aquarium (PDZA). During 2003–2008 these organizations conducted three independent tagging operations on sixgills in Puget Sound. Data acquired through these efforts included the capture and tagging of over 300 sharks in and revealed many aspects of their presence and movements in Puget Sound [10]–[12].

One of the most interesting findings of this collaborative research was that all of the sixgills caught in Puget Sound were found to be subadults [12], [13]. Average size for males was 2.4 m (range: 1.5–2.9 m) while the average size for females was 2.5 m (range: 1.7–3.1 m), smaller than estimated sizes of maturity for both sexes [6], [9], [12], [13]. Acoustic tagging and tracking of a subset of these sharks caught revealed both short term and long term movement patterns. Short term movements of acoustically tagged sharks monitored by active tracking of individuals for 24–48 hours revealed diel vertical migration patterns [10]–[12]. These sharks were found to make vertical migrations at dawn and dusk, being found in shallower waters at night (25–141 m) and deeper waters during the day (42–170 m) [11]. Long term movement patterns of sixgills using both active and passive tracking revealed sixgills exhibiting sedentary behavior with restricted daily movements and high site fidelity to the area in which they were caught and tagged [7], [14]. However, these sharks were not limited to just one area and shifted seasonally every six months between summer areas and winter areas [7], [14]. These adjacent resident areas were approximately 10–25 km apart with the summer area to the north of the winter area [7], [14]. This apparent seasonal shift between resident areas corroborated an earlier study of sixgills in the shallow (40 m) inland waters of Georgia Strait in British Columbia, Canada [15]. Between 2001–2002, using video camera data, Dunbrack and Zielinski (2003) documented strong seasonality of sixgill presence with significantly greater abundance of sharks during the summer months relative to the rest of the year [15].

Here we report the results of the SA research efforts from 2003–2005 using video analysis, external tagging, and mark-resight statistical techniques in Elliott Bay. The null hypothesis was that numbers of sharks observed at the SA research station did not change over the study period or seasonally. Research questions were as follows: Could we determine relative abundance of sixgills in Elliott Bay using video analysis techniques and mark-resight statistical methods? And were sixgills more abundant in Elliott Bay during the summer than in the winter as suggested by local diver sightings?

## Materials and Methods

The SA does not have an Institutional Animal Care and Use Committee (IACUC). The SA has an equivalent in-house animal research advisory committee, the Seattle Aquarium Research Center for Conservation and Husbandry (SEARCCH), made up of 10 outside researchers at the PhD level in addition to the SA’s director and curators. The SEARCCH committee approved this research before tagging commenced in 2003. In addition a permit from WDFW for the external tagging and biopsy of up to 50 sixgill sharks per year was obtained annually between 2003–2005 (WDFW scientific Collection Permit numbers 03-040, 04-036, and 05-036a).

**Figure 1:**
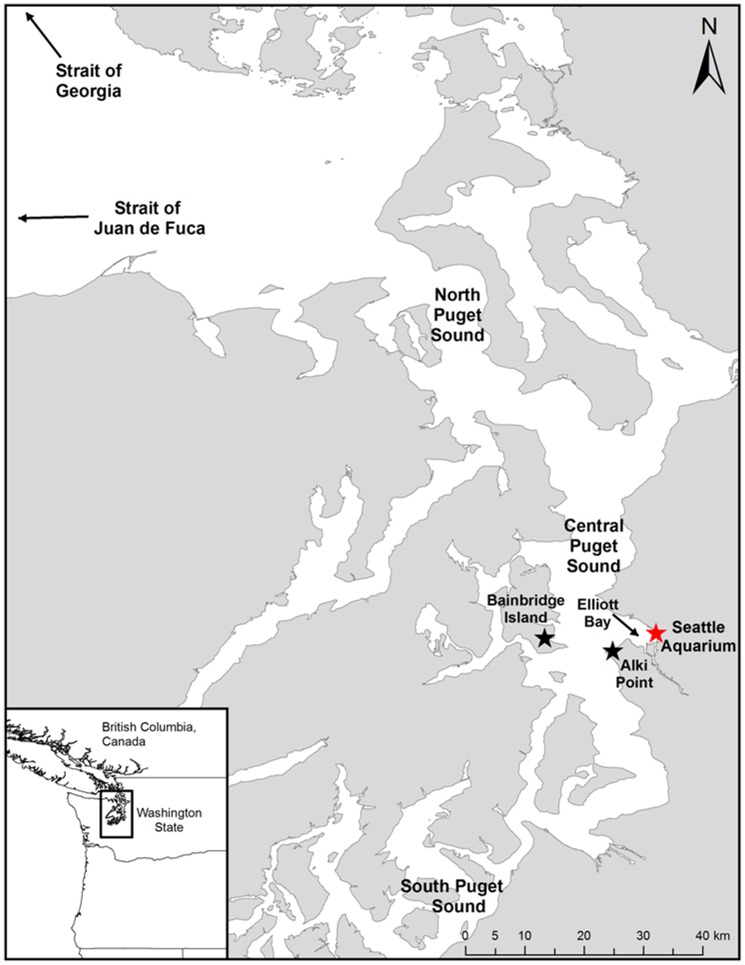
Map of Puget Sound. The location of the Seattle Aquarium is marked with a red star.

Puget Sound is a large, fjord-like estuary comprising most of the northwestern quarter of Washington State (Figure 1) [16], [17]. Elliott Bay is situated on the east side of central Puget Sound’s main basin and the SA is centrally located in Seattle’s waterfront on Piers 59 and 60 in Elliott Bay (Figure 1). The sixgill shark research site is located underneath the west end of Pier 59 in 20 m of water that leads to a steep drop-off in excess of 150 m [16].

A protected contact caged area under the SA was constructed to provide a permanent protective barrier for research divers during shark tagging operations. The cage was constructed by wrapping vinyl-coated wire fencing around seven pilings. This area was approximately 3 m^2^, and enclosed on five sides with openings on the top and east sides to allow diver access and egress during video observations and tagging/biopsy procedures. A bait station was placed 1 m west of the cage. The research area was surrounded by four fixed lights (2-Super-SeaLite and 2-Multi-SeaLite; Deep Sea Power & Light, San Diego, CA), and five fixed cameras (3-Delta Vision Industrial and 2-Deep Blue Pro; Ocean Systems Inc., Everett, WA) for video documentation of shark behavior.

Bait was used to attract sharks to the research station. The bait was placed in an open top, fiberglass reinforced plastic grated box. Divers placed up to 80 l of thawed bait in the bait box and one to three 'chumsicles' attached via 1 m stainless steel anchors. The chumsicle was made using the same bait as that placed in the bait box, but was frozen to a tether in 20 l buckets. The frozen chumsicles floated suspended above the bait station by the tether to provide a scent attractant higher in the water column with continual dispersal of scent as it thawed. Bait typically consisted of salmon (*Oncorhynchus sp)* and halibut (*Hippoglossus stenolepis*) carcasses although other species were occasionally present including Pacific herring (*Clupea pallasi*), Pacific spiny dogfish (*Squalus acanthias*), skilfish (*Erilepis zonifer*), and giant Pacific octopus (*Enteroctopus dofleini*).

The SA study used a consistent bimonthly level of effort year-round from January 2003 through May 2005 on odd months (January, March, May, July, September, and November) with additional research events in June 2003 and April 2004, for a total of 17 events. Research events comprised a four-day period with site set-up and camera installation on day one, research activities on nights two and three, and site take down on day four. Research activities began on the evening of night two with bait placement at 18:00, with bait typically refreshed 24 hours later (18:00 on night three). Research events were one to two nights in duration, with only four of the total 17 events of one night duration.

**Figure 2:**
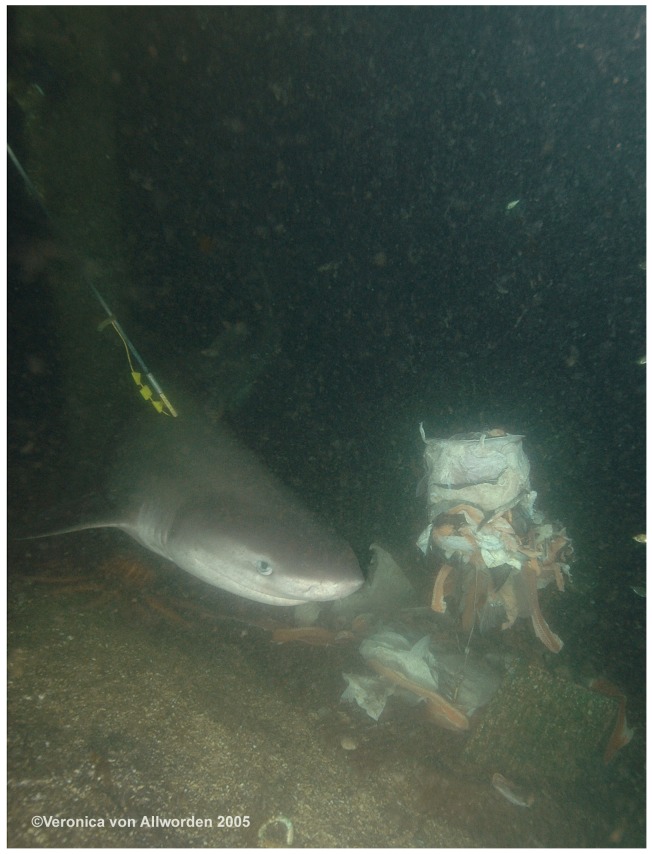
Picture of a shark swimming between the protected contact diver cage (not pictured on the left) and the bait box (on the right) while an external tag is being implanted via pole spear by SCUBA diver at the SA research station.

This research involved videotaping the sharks and implanting visual marker tags. Animals were tagged in situ by SCUBA divers while free swimming by the research station. Sharks were tagged using visual tags (Floy VM69 stainless-steel dart tags) extended in length to 30 cm and modified to contain unique shape combinations for individual identification of sharks. Four different plastic shapes (circle, square, triangle, and cross), each approximately 2.5 cm square, were attached to the streamer in up to four locations to yield 256 possible tag combinations. The tags were also imprinted with SA contact information in the event of retrieval by divers or fishermen. Divers attached the visual tags using a pole spear as sharks approached during baiting operations. The dart tags were inserted into the dorsal musculature anterior to the dorsal fin and inserted at an angle towards the head end of the shark using standard methodology (Figure 2) [18].

All shark observations and SCUBA diver activity (tagging and biopsy attempts) were documented on video tape. Video was recorded for 12 hours per night (from 18:00 to 06:00 the following morning) and event dates were defined from start to end of video recording. The video recordings were analyzed to note presence/absence of sixgills, to identify individual sixgills, to determine sex and to record tag status [19]. The goal was to determine total number of recognizable individual sharks per event to estimate abundance and number of returns. Each shark observation was documented and defined as the period of time when an individually recognized shark was visible on the video (Table S1).

Individual sharks were identified using two methods; visual tag shape sequence and unique morphological characteristics. Some sixgills had numerous marks with distinctive patterns. One challenge in identifying individual sixgills was that these marks could occur anywhere on the body in contrast to photo ID techniques used on great white sharks that tend to concentrate on one part of the body such as the dorsal fin [20]. In addition these marks could change over time due to new injuries or healing of old ones [20]. For this study, the assumption was made that an individual animal’s marks would remain static for the duration of a single research event (i.e., 2 days). However we do not assume the marks persist between events. Thus untagged sharks (UT, Table S1) when seen between events were given a new id number even if they may have been seen before. Only tagged sharks (T, Table S1) were not given a new ID number and instead were counted as a return of a previously identified shark.

To count individual sharks during the video analysis, sharks were denoted either as tagged or untagged. The tag site, tag shape sequence, sex, and unique markings were recorded from the video sighting (see Video S1) on a line diagram showing three views of a generic sixgill: Right side, dorsal side, and left side. The preferred method for identifying an individual was the diver-implanted visual marker tag (Figure 2). However, when a sixgill was untagged or the tag shape sequence could not be seen, then additional factors were used for identification such as tag location, sex, and markings. While photographs of both sides of an individual sixgill would be optimal, it was rare to view the entire sixgill in a single video frame due to the shark’s size and limited water visibility. If at least one side of a sixgill, right or left, was seen and the shark’s markings did not match up with any known sixgills, then a new identification number was assigned indicating a potentially unique individual (Table S1) [19]. Over the course of a research event, these "orphaned sides" were resolved when subsequent footage revealed both sides of an individual. Since the identifiability of a shark varied based on factors such as lighting, distance from camera, and the shark’s direction of travel, each observation was given an “ID confidence” variable with levels of “confident,” “tentative,” or “unidentifiable” for an untagged shark. Sharks with three or more identifiable markings are given a “confident” identification level, and sharks with some amount of marking less than three identifiable marks are generally “tentative.”

In addition to reporting absolute numbers of tagged and untagged sharks seen for each research event, the number of sharks was also analyzed using a Mark-Resight statistical model. In the Mark-Resight paradigm, researchers introduce some field-readable marks into the population (external visual tags in this case) and then collect encounter data (via sightings) on both the marked and unmarked individuals (i.e., tagged and untagged) in the population [21]–[23]. Of the three Mark-Resight (M-RS) models described by White and Cooch 2012, only the Zero-truncated Poisson log-normal model (ZPNE) does not assume we know the number of tagged sharks in the population at all times [21]–[23], a condition we knew we could not meet. This model is robust in that the likelihood of encountering a tagged shark is the product of two likelihoods, the “open” (primary) likelihood and the “closed” (secondary) [23]. For this dataset, the “open” or primary sessions corresponded to research events (i.e. a two-day data collection period) and the “closed” or secondary sessions corresponded to unique dates within an event. The design is based on the assumption that during a single primary session the population is totally closed because the time period is too short for temporary emigration (leaving the sampling area but with the possibility of returning) or permanent emigration (deaths or otherwise leaving with no chance of returning) to occur. However during the time between primary sessions we expect the population to be open during which permanent and temporary emigration may occur.

Based on the nature of the sixgill observation data there may be several violations of ZPNE model assumptions. The most fundamental assumption of the model was that the tagged and untagged sharks behaved identically and were thus seen identically. This means that the divers did not selectively tag sharks in some way such that the tagged sharks had different characteristics than the untagged ones, and that the tags did not affect the observability of the sharks on the video. The former assumption seems to hold, but the latter does not. Tags clearly increase a shark’s observability on the video, especially if the shark did not have other unique markings. Because this is an important assumption of the model, we addressed it as follows: We revisited all the videotape for tagged shark sightings, and recorded a new variable, “equivalency” (short for “tagged equivalency to untagged”) by evaluating the shark’s identifiability based on other unique markings as if it had been untagged. When we added this data into the model, we excluded all tagged sharks that had “tentative” or “unidentifiable” equivalency (resulting in four sharks being excluded).

Next we had to deal with the sharks seen from a single side (“orphaned side” sightings). Including them in the model could lead to overestimates of abundance if, for example, a right-sided orphaned side and a left-sided orphaned side were actually the same individual, termed potential duplicate (PD) in Table S1. We could have chosen to include left-sided orphaned sides only, but for some events, there may have been more right-sided orphaned sides. Instead we evaluated the orphaned sides in each event and made individual determinations as to whether or not they were potential duplicates of other orphaned sides. Because the dataset is limited we opted to run the program twice, once including all orphaned-sides, maximal, and again excluding the potential duplicates, minimal (Table 1 and Table S1).

**Table 1:** Mark-resight model parameter estimates, and 95% lower (LCL) and upper (UCL) confidence levels for abundance parameters for 10 primary sessions.

		Include	PD		Exclude	PD	
event number	Date	N	LCL	UCL	N	LCL	UCL
4	Jun-03	68	44	105	62	41	97
5	Jul-03	88	58	132	82	55	124
6	Sep-03	68	44	105	63	41	97
8	Jan-04	42	17	101	37	15	88
9	Mar-04	32	13	81	27	11	68
11	May-04	65	42	102	60	39	93
12	Jul-04	98	65	146	92	62	138
15	Jan-05	33	13	81	27	11	68
16	Mar-05	51	22	122	46	20	108
17	May-05	75	49	115	70	46	107

Note: Include PD = includes all orphaned sides noted in Table S1 including R-PD and L-PD. Exclude PD means includes only R and L orphaned sides that are not PD.

The significance of differences in the number of sharks seen between seasons and years was evaluated using Mann Whitney U tests with Bonferonni corrected significance level p<0.01. The significance between observed and expected sex ratio was determined using chi-square analysis.

## Results

**Figure 3:**
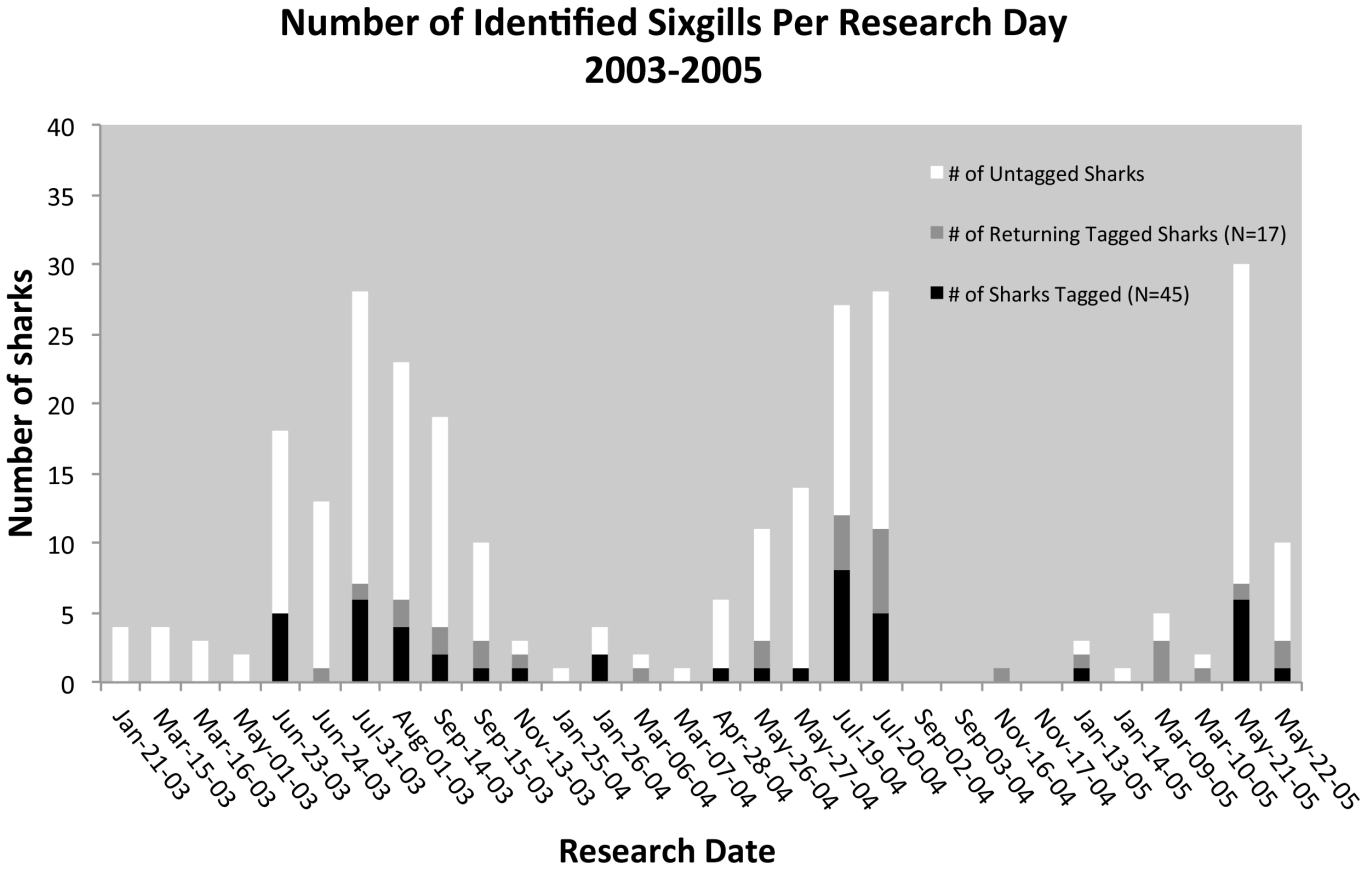
Number of Sixgills Identified on Each Research Day by Sighting Type (Untagged Shark, Newly Tagged Shark, Returning Tagged Shark).

Sixgills were seen at all SA research events via video from 2003–2005, except for during September 2004 (Table 2, Figure 3 and Table S1). The total number of observations was 273 (45 tagged, 197 untagged, and 31 returning tagged-Table 2) excluding orphaned side sharks which are potential duplicates (Table S1). The daily count ranged from a low of 0 on September 2–3, 2004 and on November 17, 2004 to a high of 30 on May 21, 2005 (Table 2).

**Table 2:** Number of sharks observed at Seattle Aquarium by research date.

Date	N	Untagged	New Tags	Tagged Returns	N of tags at liberty	Season
**1/21/2003**	4	4	0	0	0	L
**3/15/2003**	4	4	0	0	0	L
**3/16/2003**	3	3	0	0	0	L
**5/1/2003**	2	2	0	0	0	H
**6/23/2003**	18	13	5	0	0	H
**6/24/2003**	13	12	0	1	5	H
**7/31/2003**	28	21	6	1	5	H
**8/1/2003**	23	17	4	2	11	H
**9/14/2003**	19	15	2	2	15	H
**9/15/2003**	10	7	1	2	17	H
**11/13/2003**	2	0	1	1	18	L
**1/25/2004**	1	1	0	0	19	L
**1/26/2004**	4	2	2	0	19	L
**3/6/2004**	2	1	0	1	21	L
**3/7/2004**	1	1	0	0	21	L
**4/28/2004**	6	5	1	0	21	H
**5/26/2004**	11	8	1	2	22	H
**5/27/2004**	14	13	1	0	23	H
**7/19/2004**	27	15	8	4	24	H
**7/20/2004**	28	17	5	6	32	H
**9/2/2004**	0	0	0	0	37	H
**9/3/2004**	0	0	0	0	37	H
**11/16/2004**	1	0	0	1	37	L
**11/17/2004**	0	0	0	0	37	L
**1/13/2005**	3	1	1	1	37	L
**1/14/2005**	1	1	0	0	38	L
**3/9/2005**	5	2	0	3	38	L
**3/10/2005**	1	1	0	0	38	L
**5/21/2005**	30	23	6	1	38	H
**5/22/2005**	11	7	1	3	44	H
**Totals**	**273**	**197**	**45**	**31**		

Note: These numbers exclude potential duplicate sharks (See Table S1). N = total number of recognizable individual sharks per event. Untagged = number of untagged sharks identified. New Tags = number of sharks tagged during a research day. Returns: number of sharks that were seen with tags placed on an earlier date. N of tags at liberty: the number of previously tagged sharks at liberty which can return. Season = L = low season (November-March) and H = high season (April-September).

Several sharks, both tagged and untagged, returned to the research station. Tagged sharks returned 31 times at various times at liberty (Table S1). Seventeen of the tagged sixgills returned one or more times resulting in a 37.8% return rate, and recreational divers submitted a sighting report for HGSA-0020 at another location in Elliott Bay, increasing the return rate to 40.0%. Some tagged sixgills never returned (N = 28) while others returned multiple times (N = 9) (Table S1). Most of the tag returns occurred the next research day (N = 12). The mean time at liberty between initial tagging and the first return was 77 days. The longest time at liberty between initial tagging and first tag return sighting was 249 days (Table S1, shark ID# HGSA-0136) and the longest time at liberty between sightings was 354 calendar days (Table S1, shark ID# HGSA-0015 for the third return). In addition there was some evidence of external tag loss. Video review provided evidence that shark ID# HGSA-0137 (initially tagged on June 23, 2003) subsequently returned on November 13, 2003 without a visual marker tag. This individual’s markings were consistent between sightings, and upon return, the sixgill had a puncture mark at the former tag site.

The abundance of sixgills estimated using the Mark-Resight model ranged from a low of 27 (95% CI range 11–68) in March 2004 (excluding potential duplicates) to high of 98 (95% CI range 65–146) in July 2004 (including potential duplicates, Table 1). Not all 17 events contributed data to the model. Seven events were excluded because either no tagging took place (events 1–3), the events were less than the normal 2 days sampling duration (events 7 and 10) and thus there was no possibility for a shark to be resighted, or because the paucity of observations would skew the model (events 13 and 14, Table 2). Finally since the data exhibited strong seasonality, we needed to incorporate that aspect into the model. We did this by binning both secondary and primary variables into “summer” and “winter” categories. The summer category or high season occurred during events 4, 5, 6, 11, 12 and 17, and the winter category or low season comprised the remaining events: 8, 9, 15 and 16 (Table 2).

Absolute and estimated numbers of sixgills showed significant seasonal differences with more sixgills observed during the summer months than in the winter months (Z value = 3.22 and p = 0.0012; U = 34 and p<0.01, see Table 2, Figure 3). However there were no significant differences between years (Z value ranged from –1.59 to 0.80, p values ranged from 0.11 to 0.42, and U ranged from 43.5 to 24.5 and was non-significant, see Table 2 and Figure 3).

Sex of the sharks was determined from the video. Of the 273 sharks, 137 were identified as females, 70 were males, and the sex could not be determined for the remaining 66 sharks (76% sexed). A clear view of the pelvic region was required in order to determine the sex of a shark. The ratio of sharks with known sex to unknowns was 3:1 and the sex ratio for females to males was 1.96:1. This sex ratio was significantly different from the 1:1 expected ratio with females more numerous at the SA research site than males (chi-square = 10.85, p<0.001).

## Discussion

This is the first study to report abundance of sixgills within Elliott Bay and Puget Sound. The levels of sixgill abundance at the SA research station reported here is likely an underestimate because it includes the population of marked sharks only. This is because only sharks with a certain amount of identifiable markings could be uniquely identified and included in the data. Sharks without markings were in fact seen on video but were excluded from our data because they could not be confidently re-identified. Thus their numbers could expand or contract within the local population without affecting our estimates. If we had an estimate of the proportion of unmarked sharks to marked sharks then we could use the model to estimate the abundance of all sharks in our study area.

We do not know how far or wide we were attracting sharks to the SA research station. However we do know most of the sharks tracked in Puget Sound were found to make small daily movements, 0.2 to 3.1 km, with the maximum displacement between acoustic detections being 29.2 km [10]. Since Elliott Bay is 21 km^2^ and approximately 9.6 km wide at the mouth, we were likely attracting sharks that are resident within Elliott Bay. However residency in Elliott Bay was thought to be seasonal [7]. Andrews et al., 2010 documented sixgills acoustically tagged and caught in Elliott Bay in the summer were found about 10 km to the southwest in the deeper channels off of Alki Point in the winter [7]. If the sharks at the SA research site behaved the same way, then perhaps the reason why we observed significantly fewer sixgills in the winter or low season was because most of them shifted to a winter home range far enough from the SA research station such that they were no longer attracted to it.

This is not the first or only study to estimate sixgill abundance from video analysis. A similar study was conducted from 2001–2007 at Flora Islets in the inland waters of southern Strait of Georgia, Canada [15], [24], [25]. At this site only sixgills swimming towards a time lapse camera or inbound were counted and thus the data reported was number of inbound sharks per hour [24]. Similar to the data reported here, they documented seasonality with many more shark sightings in the summer than at other times of the year, with a high count of up to 7 sharks per hour in late June and early July 2002 [24]. Lengths were also estimated from the video for the period of 2001–2002 when sixgills were most abundant and all sharks observed were found to be subadults, similar to the finding of sharks in Puget Sound [25]. Dunbrack and Zielinski (2005) identified a total of 35 individuals over the two summer seasons, 2001–2002 [25]. These abundance estimates in the Flora Islets are relatively low compared to estimates reported here in Elliott Bay: 80 individuals in the summer of 2003, and 55 individuals in summer 2004 (Table 2). In addition, over the seven year study in the Flora Islets the frequency of sharks viewed in 2001 was significantly higher than in any other subsequent year with steadily decreasing observations over time, with the sharks observed in 2006 only 1% of those seen in 2001 [15].

The video analysis techniques used here had limitations and unfortunately were not designed to accurately size sharks. However sixgills sampled in Puget Sound during 2003–2007 via longline by our research partners (WDFW and NOAA NMFS) were found to be exclusively subadults, or males less than 3 m and females less than 4 m [12], [13]. Thus we assumed that the sharks observed at the SA research station were also subadults. In addition, genetic studies of sharks sampled at the SA research station and by our research partners revealed a high degree of relatedness (full and half siblings) among sixgills sampled at the same time and place (such as one longline set or SA research event), suggesting subadult cohorts may travel together [13]. Thus the sixgills sampled at the SA research station may have been the resident cohort group that lived in Elliott Bay in the summer. This cohort group likely then shifted to their southern resident range in the winter resulting in the lower numbers of sixgills observed during the low or winter season (Table 1, 2 and Figure 3).

Sex was easier to determine from video observations than size. Female sixgills were observed significantly more often than males at the SA research site. Sixgills have not been previously documented as sexually segregating; although sexual segregation has been reported in other shark species including the scalloped hammerhead shark, *Sphyrna lewini* [26], [27], the great white shark [28], the lemon shark *Negaprion brevirostris* [29], and the blue shark [30]. However the sex ratio of sixgills sampled by our research partners in Puget Sound did not differ significantly from 1:1 [12]. We do not know whether female sixgills were preferentially attracted to our research site, if the resident sixgills in the area were predominately female, or if the sex ratio was skewed due to the difficulty of sexing young male sharks via video analysis.

More research needs to be done to determine if this apex predators’ subadult residence to the inland waterways of the Northeast pacific is unique. While the observed pattern of seasonal shifts in sixgill abundance has also been reported in other cowsharks, such as broadnose sevengills [9], [31], the factors driving these seasonal changes may differ. For example, Lucifora et al. (2005) theorized that shallow bays serve as nursery areas for sevengills in Patagonia, Argentina [32] while Williams et al. 2010 suggested that sevengills may use Pacific Northwest estuaries for foraging [12]. In Puget Sound, Andrews et al. (2010) proposed that seasonality in sixgill abundance may be prey driven [7]. Sixgills are thought to utilize both scavenging and active predation, however, the relative importance of these feeding strategies is unknown [9]. The spotted ratfish, *Hydrolagus colliei*, and Pacific spiny dogfish are thought to be major prey items for Puget Sound sixgills [33]. Quinn et al. (1980) found *H. colliei* exhibiting similar seasonal shifts to shallower waters in spring and summer followed by movements to deeper water in fall and winter [34]. In addition, Reum and Essington (2008) found that Pacific spiny dogfish were more evident in Puget Sound in the summer and fall than in winter [35]. Thus perhaps the seasonality of sixgill abundance at the SA research site in Elliott Bay were driven by the resident sixgills following their preferred prey.

## Supporting Information

Table S1 **List of individual sharks observed at Seattle Aquarium by research date.**
(DOCX)

Video S1 **Video of the right side of shark ID #HGSA-0265.**
(MPG)
