# Supplementary material for: Observations on Abundance of Bluntnose Sixgill Sharks, Hexanchus griseus, in an Urban Waterway in Puget Sound, 2003-2005
Source: PLoS One. 2014 Jan 27;9(1):e87081. doi: 10.1371/journal.pone.0087081 (PMC3903586; doi:10.1371/journal.pone.0087081)
Supplement: Table S1 — List of individual sharks observed at Seattle Aquarium by research date. (DOCX) [file pone.0087081.s001.docx]

Appendix 1: List of individual sharks observed at Seattle Aquarium by research date

| Research Date | Event ID | Day Number | Shark ID | Sex | Sighting Type | Tag Shape Sequence | ID Confidence | Equivalency | Orphaned Side - Potential Duplicate | Return Dates |
| --- | --- | --- | --- | --- | --- | --- | --- | --- | --- | --- |
| 01/21/03 | 1 | 1 | HGSA-0128 | M | UI |  | C | U |  | None |
| 01/21/03 | 1 | 1 | HGSA-0129 | F | UI |  | C | U |  | None |
| 01/21/03 | 1 | 1 | HGSA-0130 | M | UI |  | C | U |  | None |
| 01/21/03 | 1 | 1 | HGSA-0131 | U | UI |  | T | U | R | None |
| 03/15/03 | 2 | 1 | HGSA-0158 | F | UI |  | C | U |  | 03/16/03* |
| 03/15/03 | 2 | 1 | HGSA-0159 | M | UI |  | C | U |  | None |
| 03/15/03 | 2 | 1 | HGSA-0164 | M | UI |  | C | U |  | 03/16/03* |
| 03/15/03 | 2 | 1 | HGSA-0171 | F | UI |  | C | U |  | None |
| 03/16/03 | 2 | 2 | HGSA-0158 | F | UR |  | C | U |  |  |
| 03/16/03 | 2 | 2 | HGSA-0164 | M | UR |  | C | U |  |  |
| 03/16/03 | 2 | 2 | HGSA-0192 | M | UI |  | C | U | R | None |
| 05/01/03 | 3 | 1 | HGSA-0132 | U | UI |  | C | U | R-PD | None |
| 05/01/03 | 3 | 1 | HGSA-0133 | F | UI |  | C | U |  | None |
| 05/01/03 | 3 | 1 | HGSA-0134 | M | UI |  | C | U | R | None |
| 06/23/03 | 4 | 1 | HGSA-0013 | F | NT | SP-3-FLP | C | TC |  | 07/31/03 |
| 06/23/03 | 4 | 1 | HGSA-0014 | F | NT | SP-2-FLM | C | TC |  | 08/01/03 |
| 06/23/03 | 4 | 1 | HGSA-0015 | F | NT | SP-4-FR | S | TC |  | 6/24/03, 08/01/03, 07/20/04, 05/22/05 |
| 06/23/03 | 4 | 1 | HGSA-0034 | F | UI |  | C | U |  | None |
| 06/23/03 | 4 | 1 | HGSA-0035 | F | UI |  | C | U |  | None |
| 06/23/03 | 4 | 1 | HGSA-0036 | F | UI |  | C | U |  | 06/24/03* |
| 06/23/03 | 4 | 1 | HGSA-0037 | M | UI |  | C | U | L^$^ | None |
| 06/23/03 | 4 | 1 | HGSA-0038 | M | NT | SP-5-MR (retagged as CSC on 11/13/03) | S | TC |  | 11/13/03 |
| 06/23/03 | 4 | 1 | HGSA-0089 | M | UI |  | C | U |  | None |
| 06/23/03 | 4 | 1 | HGSA-0092 | F | UI |  | C | U |  | None |
| 06/23/03 | 4 | 1 | HGSA-0102 | M | UI |  | C | U |  | None |
| 06/23/03 | 4 | 1 | HGSA-0103 | M | UI |  | C | U |  | 06/24/03* |
| 06/23/03 | 4 | 1 | HGSA-0139 | U | UI |  | C | U |  | 06/24/03* |
| 06/23/03 | 4 | 1 | HGSA-0255 | M | NT | SP-1-ML | C | TC |  | None |
| 06/23/03 | 4 | 1 | HGSA-0263 | F | UI |  | C | U |  | None |
| 06/23/03 | 4 | 1 | HGSA-0264 | F | UI |  | C | U | R^$^ | None |
| 06/23/03 | 4 | 1 | HGSA-0265 | M | UI |  | C | U |  | None |
| 06/23/03 | 4 | 1 | HGSA-0266 | M | UI |  | C | U |  | None |
| 06/24/03 | 4 | 2 | HGSA-0015 | U | TR | SP-4-FR | S | TR |  |  |
| 06/24/03 | 4 | 2 | HGSA-0036 | U | UR |  | C | U |  |  |
| 06/24/03 | 4 | 2 | HGSA-0103 | M | UR |  | C | U |  |  |
| 06/24/03 | 4 | 2 | HGSA-0125 | F | UI |  | C | U |  | None |
| 06/24/03 | 4 | 2 | HGSA-0127 | M | UI |  | C | U |  | None |
| 06/24/03 | 4 | 2 | HGSA-0139 | F | UR |  | C | U |  |  |
| 06/24/03 | 4 | 2 | HGSA-0155 | F | UI |  | C | U |  | None |
| 06/24/03 | 4 | 2 | HGSA-0156 | U | UI |  | C | U |  | None |
| 06/24/03 | 4 | 2 | HGSA-0157 | F | UI |  | C | U |  | None |
| 06/24/03 | 4 | 2 | HGSA-0181 | F | UI |  | C | U |  | None |
| 06/24/03 | 4 | 2 | HGSA-0185 | F | UI |  | C | U |  | None |
| 06/24/03 | 4 | 2 | HGSA-0186 | U | UI |  | C | U |  | None |
| 06/24/03 | 4 | 2 | HGSA-0187 | M | UI |  | C | U |  | None |
| 07/31/03 | 5 | 1 | HGSA-0008 | M | UI |  | C | U |  | None |
| 07/31/03 | 5 | 1 | HGSA-0011 | F | NT | S | S | TC |  | None |
| 07/31/03 | 5 | 1 | HGSA-0013 | F | TR | SP-3-FLP | S | TR |  |  |
| 07/31/03 | 5 | 1 | HGSA-0016 | F | NT | C | C | TC |  | 09/14/03, 09/15/03 |
| 07/31/03 | 5 | 1 | HGSA-0021 | F | NT | SS | C | TC |  | None |
| 07/31/03 | 5 | 1 | HGSA-0022 | M | NT | CC | S | TC |  | None |
| 07/31/03 | 5 | 1 | HGSA-0023 | F | UI |  | C | U |  | None |
| 07/31/03 | 5 | 1 | HGSA-0027 | F | UI |  | C | U |  | 08/01/03* |
| 07/31/03 | 5 | 1 | HGSA-0028 | U | UI |  | C | U |  | None |
| 07/31/03 | 5 | 1 | HGSA-0029 | M | UI |  | C | U |  | None |
| 07/31/03 | 5 | 1 | HGSA-0030 | M | NT | SC | C | TC |  | None |
| 07/31/03 | 5 | 1 | HGSA-0032 | F | NT | CS | C | TC |  | None |
| 07/31/03 | 5 | 1 | HGSA-0033 | F | UI |  | C | U |  | 08/01/03* |
| 07/31/03 | 5 | 1 | HGSA-0043 | F | UI |  | C | U |  | 08/01/03 |
| 07/31/03 | 5 | 1 | HGSA-0061 | U | UI |  | T | U |  | None |
| 07/31/03 | 5 | 1 | HGSA-0063 | F | UI |  | C | U |  | 08/01/03* |
| 07/31/03 | 5 | 1 | HGSA-0064 | F | UI |  | C | U |  | None |
| 07/31/03 | 5 | 1 | HGSA-0081 | U | UI |  | C | U |  | None |
| 07/31/03 | 5 | 1 | HGSA-0083 | M | UI |  | C | U |  | None |
| 07/31/03 | 5 | 1 | HGSA-0084 | F | UI |  | C | U |  | None |
| 07/31/03 | 5 | 1 | HGSA-0085 | U | UI |  | C | U |  | None |
| 07/31/03 | 5 | 1 | HGSA-0086 | F | UI |  | C | U |  | 08/01/03* |
| 07/31/03 | 5 | 1 | HGSA-0087 | F | UI |  | C | U |  | 08/01/03* |
| 07/31/03 | 5 | 1 | HGSA-0088 | F | UI |  | C | U |  | None |
| 07/31/03 | 5 | 1 | HGSA-0116 | M | UI |  | C | U |  | None |
| 07/31/03 | 5 | 1 | HGSA-0117 | U | UI |  | C | U | L | None |
| 07/31/03 | 5 | 1 | HGSA-0196 | U | UI |  | C | U |  | None |
| 07/31/03 | 5 | 1 | HGSA-0202 | U | UI |  | C | U | L | None |
| 08/01/03 | 5 | 2 | HGSA-0014 | F | TR | SP-2-FLM | S | TR |  |  |
| 08/01/03 | 5 | 2 | HGSA-0015 | F | TR | SP-4-FR | S | TR |  |  |
| 08/01/03 | 5 | 2 | HGSA-0027 | U | UR |  | C | U |  |  |
| 08/01/03 | 5 | 2 | HGSA-0033 | F | UR |  | C | U |  |  |
| 08/01/03 | 5 | 2 | HGSA-0043 | F | UR, NT | CCC | S | TC |  | None |
| 08/01/03 | 5 | 2 | HGSA-0056 | F | UI |  | C | U |  | None |
| 08/01/03 | 5 | 2 | HGSA-0057 | F | UI |  | C | U |  | None |
| 08/01/03 | 5 | 2 | HGSA-0058 | F | UI |  | C | U |  | None |
| 08/01/03 | 5 | 2 | HGSA-0063 | F | UR |  | C | U |  |  |
| 08/01/03 | 5 | 2 | HGSA-0086 | U | UR |  | T | U |  |  |
| 08/01/03 | 5 | 2 | HGSA-0087 | U | UR |  | T | U |  |  |
| 08/01/03 | 5 | 2 | HGSA-0095 | F | NT | SSS | S | TC |  | 09/14/03, 09/15/03 |
| 08/01/03 | 5 | 2 | HGSA-0097 | M | UI |  | C | U |  | None |
| 08/01/03 | 5 | 2 | HGSA-0098 | M | UI |  | C | U |  | None |
| 08/01/03 | 5 | 2 | HGSA-0100 | F | NT | SCC | S | TC |  | None |
| 08/01/03 | 5 | 2 | HGSA-0108 | M | NT | SSC | S | TC |  | None |
| 08/01/03 | 5 | 2 | HGSA-0109 | U | UI |  | C | U |  | None |
| 08/01/03 | 5 | 2 | HGSA-0110 | M | UI |  | C | U | L | None |
| 08/01/03 | 5 | 2 | HGSA-0111 | U | UI |  | C | U | L | None |
| 08/01/03 | 5 | 2 | HGSA-0112 | U | UI |  | C | U | R-PD | None |
| 08/01/03 | 5 | 2 | HGSA-0118 | M | UI |  | C | U |  | None |
| 08/01/03 | 5 | 2 | HGSA-0119 | U | UI |  | C | U | R-PD | None |
| 08/01/03 | 5 | 2 | HGSA-0120 | U | UI |  | C | U |  | None |
| 08/01/03 | 5 | 2 | HGSA-0123 | U | UI |  | C | U | L | None |
| 08/01/03 | 5 | 2 | HGSA-0124 | F | UI |  | C | U | L | None |
| 09/14/03 | 6 | 1 | HGSA-0016 | F | TR | C | S | TR |  |  |
| 09/14/03 | 6 | 1 | HGSA-0095 | F | TR | SSS | S | TR |  |  |
| 09/14/03 | 6 | 1 | HGSA-0148 | M | UI |  | C | U |  | None |
| 09/14/03 | 6 | 1 | HGSA-0150 | U | UI |  | C | U | L | 09/15/03* |
| 09/14/03 | 6 | 1 | HGSA-0160 | F | UI |  | C | U |  | None |
| 09/14/03 | 6 | 1 | HGSA-0162 | F | UI |  | C | U |  | None |
| 09/14/03 | 6 | 1 | HGSA-0168 | U | UI |  | C | U |  | 09/15/03* |
| 09/14/03 | 6 | 1 | HGSA-0169 | U | UI |  | C | U |  | None |
| 09/14/03 | 6 | 1 | HGSA-0170 | F | UI |  | C | U |  | None |
| 09/14/03 | 6 | 1 | HGSA-0173 | F | UI |  | C | U |  | None |
| 09/14/03 | 6 | 1 | HGSA-0174 | U | UI |  | C | U | L | None |
| 09/14/03 | 6 | 1 | HGSA-0176 | U | UI |  | C | U |  | None |
| 09/14/03 | 6 | 1 | HGSA-0177 | M | UI |  | C | U |  | None |
| 09/14/03 | 6 | 1 | HGSA-0189 | F | NT | CSS | S | TC |  | None |
| 09/14/03 | 6 | 1 | HGSA-0193 | F | UI |  | C | U |  | None |
| 09/14/03 | 6 | 1 | HGSA-0194 | U | UI |  | C | U |  | 09/15/03* |
| 09/14/03 | 6 | 1 | HGSA-0198 | F | UI |  | C | U |  | 09/15/03* |
| 09/14/03 | 6 | 1 | HGSA-0199 | U | UI |  | C | U |  | 09/15/03* |
| 09/14/03 | 6 | 1 | HGSA-0276 | F | NT | CCS | S | TC | L | None |
| 09/15/03 | 6 | 2 | HGSA-0016 | F | TR | C | S | TR |  |  |
| 09/15/03 | 6 | 2 | HGSA-0095 | F | TR | SSS | S | TR |  |  |
| 09/15/03 | 6 | 2 | HGSA-0150 | F | UR |  | T | U | L |  |
| 09/15/03 | 6 | 2 | HGSA-0168 | F | UR, NT | SCS | S | TC |  | None |
| 09/15/03 | 6 | 2 | HGSA-0194 | U | UR |  | C | U |  |  |
| 09/15/03 | 6 | 2 | HGSA-0198 | F | UR |  | C | U |  |  |
| 09/15/03 | 6 | 2 | HGSA-0199 | M | UR |  | C | U |  |  |
| 09/15/03 | 6 | 2 | HGSA-0212 | F | UI |  | C | U |  | None |
| 09/15/03 | 6 | 2 | HGSA-0216 | F | UI |  | C | U | R-PD | None |
| 09/15/03 | 6 | 2 | HGSA-0219 | M | UI |  | C | U |  | None |
| 09/15/03 | 6 | 2 | HGSA-0221 | U | UI |  | C | U |  | None |
| 11/13/03 | 7 | 1 | HGSA-0038 | M | TR, NT | CSC (formerly SP-5-MR) | C | TT |  |  |
| 11/13/03 | 7 | 1 | HGSA-0136 | F | NT | CSSS | S | TC |  | 07/19/04 |
| 11/13/03 | 7 | 1 | HGSA-0147 | U | UI |  | C | U | L | None |
| 01/25/04 | 8 | 1 | HGSA-0146 | M | UI |  | C | U | L | None |
| 01/26/04 | 8 | 2 | HGSA-0138 | F | NT | SSSC | S | TC |  | 03/06/04,  05/26/04,  07/19/04 |
| 01/26/04 | 8 | 2 | HGSA-0141 | F | NT | CSCS | C | TC |  | 05/26/04,  07/19/04,  07/20/04, 03/09/05 |
| 01/26/04 | 8 | 2 | HGSA-0142 | F | UI |  | C | U |  | None |
| 01/26/04 | 8 | 2 | HGSA-0143 | U | UI |  | T | U | R-PD | None |
| 01/26/04 | 8 | 2 | HGSA-0144 | F | UI |  | C | U | R-PD | None |
| 01/26/04 | 8 | 2 | HGSA-0145 | F | UI |  | C | U | L | None |
| 03/06/04 | 9 | 1 | HGSA-0138 | F | TR | SSSC | S | TR |  |  |
| 03/06/04 | 9 | 1 | HGSA-0191 | F | UI |  | C | U |  | 03/07/04* |
| 03/07/04 | 9 | 2 | HGSA-0191 | U | UR |  | C | U |  |  |
| 04/28/04 | 10 | 1 | HGSA-0262 | M | UI |  | C | U |  | None |
| 04/28/04 | 10 | 1 | HGSA-0302 | M | NT | CSSC | S | TC | L^$^ | None |
| 04/28/04 | 10 | 1 | HGSA-0304 | M | UI |  | C | U | R | None |
| 04/28/04 | 10 | 1 | HGSA-0305 | U | UI |  | C | U | L-PD | None |
| 04/28/04 | 10 | 1 | HGSA-0306 | F | UI |  | C | U | R | None |
| 04/28/04 | 10 | 1 | HGSA-0307 | M | UI |  | C | U |  | None |
| 04/28/04 | 10 | 1 | HGSA-0308 | M | UI |  | C | U | R | None |
| 05/26/04 | 11 | 1 | HGSA-0047 | F | NT | CSCC | C | TC |  | 07/19/04,  07/20/04 |
| 05/26/04 | 11 | 1 | HGSA-0060 | F | UI |  | C | U |  | None |
| 05/26/04 | 11 | 1 | HGSA-0069 | U | UI |  | T | U |  | None |
| 05/26/04 | 11 | 1 | HGSA-0073 | U | UI |  | T | U | L | None |
| 05/26/04 | 11 | 1 | HGSA-0075 | M | UI |  | T | U |  | None |
| 05/26/04 | 11 | 1 | HGSA-0078 | U | UI |  | T | U | L | None |
| 05/26/04 | 11 | 1 | HGSA-0101 | M | UI |  | C | U | L | None |
| 05/26/04 | 11 | 1 | HGSA-0104 | F | UI |  | C | U |  | None |
| 05/26/04 | 11 | 1 | HGSA-0138 | F | TR | SSSC | S | TR |  |  |
| 05/26/04 | 11 | 1 | HGSA-0141 | F | TR | CSCS | C | TR |  |  |
| 05/26/04 | 11 | 1 | HGSA-0203 | F | UI |  | C | U |  | 05/27/04* |
| 05/27/04 | 11 | 2 | HGSA-0079 | F | UI |  | C | U |  | None |
| 05/27/04 | 11 | 2 | HGSA-0140 | F | UI |  | C | U |  | None |
| 05/27/04 | 11 | 2 | HGSA-0153 | F | UI |  | C | U |  | None |
| 05/27/04 | 11 | 2 | HGSA-0165 | M | UI |  | C | U |  | None |
| 05/27/04 | 11 | 2 | HGSA-0166 | U | UI |  | C | U | R-PD | None |
| 05/27/04 | 11 | 2 | HGSA-0167 | F | UI |  | C | U |  | None |
| 05/27/04 | 11 | 2 | HGSA-0179 | U | UI |  | C | U | R-PD | None |
| 05/27/04 | 11 | 2 | HGSA-0180 | F | UI |  | C | U |  | None |
| 05/27/04 | 11 | 2 | HGSA-0183 | F | UI |  | C | U |  | None |
| 05/27/04 | 11 | 2 | HGSA-0195 | M | UI |  | C | U | R-PD | None |
| 05/27/04 | 11 | 2 | HGSA-0203 | F | UR |  | C | U |  |  |
| 05/27/04 | 11 | 2 | HGSA-0204 | F | UI |  | C | U |  | None |
| 05/27/04 | 11 | 2 | HGSA-0249 | F | UI |  | C | U | R-PD | None |
| 05/27/04 | 11 | 2 | HGSA-0250 | U | NT | SCCS | T | TC | R^$^ | None |
| 05/27/04 | 11 | 2 | HGSA-0252 | U | UI |  | C | U | L | None |
| 05/27/04 | 11 | 2 | HGSA-0253 | M | UI |  | C | U |  | None |
| 05/27/04 | 11 | 2 | HGSA-0254 | M | UI |  | C | U |  | None |
| 05/27/04 | 11 | 2 | HGSA-0376 | U | UI |  | T | U | R^$^ | None |
| 07/19/04 | 12 | 1 | HGSA-0001 | F | NT | SCCC | C | TC |  | None |
| 07/19/04 | 12 | 1 | HGSA-0002 | F | UI |  | C | U |  | None |
| 07/19/04 | 12 | 1 | HGSA-0006 | F | NT | SCSC | S | TC |  | None |
| 07/19/04 | 12 | 1 | HGSA-0007 | M | UI |  | C | U |  | None |
| 07/19/04 | 12 | 1 | HGSA-0017 | F | UI |  | C | U |  | 07/20/04* |
| 07/19/04 | 12 | 1 | HGSA-0018 | F | NT | CCSC | C | TC |  | [03/09/05](mailto:3-9-05@22:) |
| 07/19/04 | 12 | 1 | HGSA-0020 | F | NT | SCSS | C | TC |  | None |
| 07/19/04 | 12 | 1 | HGSA-0024 | F | NT | CCCS | S | TC |  | 07/20/04 |
| 07/19/04 | 12 | 1 | HGSA-0039 | M | UI |  | C | U |  | None |
| 07/19/04 | 12 | 1 | HGSA-0040 | F | UI |  | C | U |  | 07/20/04* |
| 07/19/04 | 12 | 1 | HGSA-0047 | F | TR | CSCC | S | TR |  |  |
| 07/19/04 | 12 | 1 | HGSA-0136 | F | TR | CSSS | C | TR |  |  |
| 07/19/04 | 12 | 1 | HGSA-0138 | F | TR | SSSC | S | TR |  |  |
| 07/19/04 | 12 | 1 | HGSA-0141 | F | TR | CSCS | S | TR |  |  |
| 07/19/04 | 12 | 1 | HGSA-0233 | U | UI |  | C | U | L | 07/20/04* |
| 07/19/04 | 12 | 1 | HGSA-0248 | M | NT | SSSS | C | TC |  | 07/20/04,  03/09/05 |
| 07/19/04 | 12 | 1 | HGSA-0274 | F | NT | CCSS | S | TC |  | 7-20-04,  5-21-05 |
| 07/19/04 | 12 | 1 | HGSA-0309 | M | UI |  | C | U |  | None |
| 07/19/04 | 12 | 1 | HGSA-0310 | U | NT | CCCC | S | TT |  | None |
| 07/19/04 | 12 | 1 | HGSA-0311 | F | UI |  | C | U |  | 07/20/04* |
| 07/19/04 | 12 | 1 | HGSA-0314 | U | UI |  | C | U | R-PD | None |
| 07/19/04 | 12 | 1 | HGSA-0315 | F | UI |  | C | U |  | None |
| 07/19/04 | 12 | 1 | HGSA-0316 | U | UI |  | C | U | L | None |
| 07/19/04 | 12 | 1 | HGSA-0318 | F | UI |  | C | U | L | None |
| 07/19/04 | 12 | 1 | HGSA-0319 | U | UI |  | C | U | R-PD | None |
| 07/19/04 | 12 | 1 | HGSA-0320 | F | UI |  | C | U | L | None |
| 07/19/04 | 12 | 1 | HGSA-0321 | F | UI |  | C | U | L | 07/20/04* |
| 07/19/04 | 12 | 1 | HGSA-0322 | M | UI |  | C | U | L | None |
| 07/19/04 | 12 | 1 | HGSA-0323 | U | UI |  | C | U | L | None |
| 07/20/04 | 12 | 2 | HGSA-0015 | F | TR | SP-4-FR | S | TR |  |  |
| 07/20/04 | 12 | 2 | HGSA-0017 | F | UR |  | C | U |  |  |
| 07/20/04 | 12 | 2 | HGSA-0024 | F | TR | CCCS | S | TR |  |  |
| 07/20/04 | 12 | 2 | HGSA-0040 | F | UR |  | C | U |  |  |
| 07/20/04 | 12 | 2 | HGSA-0047 | U | TR | CSCC | C | TR |  |  |
| 07/20/04 | 12 | 2 | HGSA-0141 | F | TR | CSCS | S | TR |  |  |
| 07/20/04 | 12 | 2 | HGSA-0233 | F | UR |  | C | U | L |  |
| 07/20/04 | 12 | 2 | HGSA-0234 | F | NT | SSCS | S | TC |  | None |
| 07/20/04 | 12 | 2 | HGSA-0235 | U | UI |  | C | U |  | None |
| 07/20/04 | 12 | 2 | HGSA-0236 | F | UI |  | C | U |  | None |
| 07/20/04 | 12 | 2 | HGSA-0237 | F | UI |  | C | U |  | None |
| 07/20/04 | 12 | 2 | HGSA-0238 | F | UI |  | C | U |  | None |
| 07/20/04 | 12 | 2 | HGSA-0245 | M | UI |  | C | U | R-PD | None |
| 07/20/04 | 12 | 2 | HGSA-0246 | M | UI |  | C | U | L | None |
| 07/20/04 | 12 | 2 | HGSA-0247 | U | UI |  | C | U |  | None |
| 07/20/04 | 12 | 2 | HGSA-0248 | M | TR | SSSS | S | TR |  |  |
| 07/20/04 | 12 | 2 | HGSA-0258 | U | UI |  | C | U | L | None |
| 07/20/04 | 12 | 2 | HGSA-0273 | F | NT | ST | S | TC |  | 01/13/05 |
| 07/20/04 | 12 | 2 | HGSA-0274 | F | TR | CCSS | S | TR |  |  |
| 07/20/04 | 12 | 2 | HGSA-0311 | F | UR |  | C | U |  |  |
| 07/20/04 | 12 | 2 | HGSA-0321 | U | UR |  | C | U | L |  |
| 07/20/04 | 12 | 2 | HGSA-0326 | M | UI |  | C | U | L | None |
| 07/20/04 | 12 | 2 | HGSA-0327 | M | UI |  | C | U |  | None |
| 07/20/04 | 12 | 2 | HGSA-0329 | F | NT | SSCC | S | TC |  | 11/16/04 |
| 07/20/04 | 12 | 2 | HGSA-0330 | M | NT | T | S | TC |  | None |
| 07/20/04 | 12 | 2 | HGSA-0331 | M | NT | TS | S | TC |  | None |
| 07/20/04 | 12 | 2 | HGSA-0332 | M | UI |  | C | U |  | None |
| 07/20/04 | 12 | 2 | HGSA-0333 | U | UI |  | C | U | L | None |
| 07/20/04 | 12 | 2 | HGSA-0336 | U | UI |  | C | U |  | None |
| 09/02/04 | 13 | 1 |  |  |  |  |  |  |  |  |
| 09/03/04 | 13 | 2 |  |  |  |  |  |  |  |  |
| 11/16/04 | 14 | 1 | HGSA-0329 | F | TR | SSCC | S | TR |  |  |
| 11/17/04 | 14 | 2 |  |  |  |  |  |  |  |  |
| 01/13/05 | 15 | 1 | HGSA-0223 | U | UI |  | C | U | R | None |
| 01/13/05 | 15 | 1 | HGSA-0273 | F | TR | ST | S | TR |  |  |
| 01/13/05 | 15 | 1 | HGSA-0338 | U | NT | TT | T | TU | R | None |
| 01/14/05 | 15 | 2 | HGSA-0277 | U | UI |  | C | U | R | None |
| 03/09/05 | 16 | 1 | HGSA-0018 | F | TR | CCSC | S | TR |  |  |
| 03/09/05 | 16 | 1 | HGSA-0141 | F | TR | CSCS | S | TR |  |  |
| 03/09/05 | 16 | 1 | HGSA-0209 | U | UI |  | C | U | L | None |
| 03/09/05 | 16 | 1 | HGSA-0248 | U | TR | SSSS | C | TR |  |  |
| 03/09/05 | 16 | 1 | HGSA-0339 | M | UI |  | C | U |  | None |
| 03/10/05 | 16 | 2 | HGSA-0215 | F | UI |  | C | U |  | None |
| 03/10/05 | 16 | 2 | HGSA-0340 | U | UI |  | C | U | R-PD | None |
| 05/21/05 | 17 | 1 | HGSA-0228 | M | UI |  | C | U |  | None |
| 05/21/05 | 17 | 1 | HGSA-0230 | M | NT | TTS | S | TC |  | None |
| 05/21/05 | 17 | 1 | HGSA-0231 | F | UI |  | C | U | L | None |
| 05/21/05 | 17 | 1 | HGSA-0232 | U | NT | TST | S | TC | R^$^ | None |
| 05/21/05 | 17 | 1 | HGSA-0242 | M | NT | TSS | S | TC |  | None |
| 05/21/05 | 17 | 1 | HGSA-0243 | U | NT | SST | S | TU | L | None |
| 05/21/05 | 17 | 1 | HGSA-0244 | F | NT | STT | S | TC |  | 05/22/05 |
| 05/21/05 | 17 | 1 | HGSA-0274 | F | TR | CCSS | S | TR |  |  |
| 05/21/05 | 17 | 1 | HGSA-0278 | F | UI |  | C | U |  | None |
| 05/21/05 | 17 | 1 | HGSA-0279 | U | UI |  | C | U |  | None |
| 05/21/05 | 17 | 1 | HGSA-0281 | U | UI |  | C | U |  | None |
| 05/21/05 | 17 | 1 | HGSA-0282 | F | UI |  | C | U |  | None |
| 05/21/05 | 17 | 1 | HGSA-0284 | F | UI |  | C | U |  | None |
| 05/21/05 | 17 | 1 | HGSA-0285 | U | UI |  | C | U | L | None |
| 05/21/05 | 17 | 1 | HGSA-0288 | F | UI |  | C | U |  | None |
| 05/21/05 | 17 | 1 | HGSA-0290 | U | UI |  | C | U |  | None |
| 05/21/05 | 17 | 1 | HGSA-0291 | F | UI |  | C | U |  | 05/22/05* |
| 05/21/05 | 17 | 1 | HGSA-0294 | U | UI |  | C | U |  | None |
| 05/21/05 | 17 | 1 | HGSA-0295 | U | UI |  | C | U | L | None |
| 05/21/05 | 17 | 1 | HGSA-0296 | F | UI |  | C | U | L | None |
| 05/21/05 | 17 | 1 | HGSA-0297 | M | UI |  | C | U |  | None |
| 05/21/05 | 17 | 1 | HGSA-0300 | U | UI |  | C | U | R-PD | None |
| 05/21/05 | 17 | 1 | HGSA-0301 | U | UI |  | C | U |  | None |
| 05/21/05 | 17 | 1 | HGSA-0328 | U | UI |  | C | U | R-PD | None |
| 05/21/05 | 17 | 1 | HGSA-0334 | M | UI |  | C | U |  | None |
| 05/21/05 | 17 | 1 | HGSA-0342 | U | UI |  | C | U | L | None |
| 05/21/05 | 17 | 1 | HGSA-0343 | M | UI |  | C | U |  | None |
| 05/21/05 | 17 | 1 | HGSA-0344 | U | UI |  | C | U | L | None |
| 05/21/05 | 17 | 1 | HGSA-0345 | U | UI |  | C | U | R-PD | None |
| 05/21/05 | 17 | 1 | HGSA-0346 | F | UI |  | C | U |  | None |
| 05/21/05 | 17 | 1 | HGSA-0347 | M | UI |  | C | U | L | None |
| 05/21/05 | 17 | 1 | HGSA-0348 | M | NT | TTT | C | TC | R^$^ | None |
| 05/21/05 | 17 | 1 | HGSA-0350 | U | UI |  | C | U | R-PD | None |
| 05/21/05 | 17 | 1 | HGSA-0351 | U | UI |  | C | U |  | None |
| 05/22/05 | 17 | 2 | HGSA-0015 | F | TR | SP-4-FR | S | TR |  |  |
| 05/22/05 | 17 | 2 | HGSA-0244 | F | TR | STT | S | TR |  |  |
| 05/22/05 | 17 | 2 | HGSA-0291 | U | UR |  | C | U |  |  |
| 05/22/05 | 17 | 2 | HGSA-0352 | M | UI |  | C | U |  | None |
| 05/22/05 | 17 | 2 | HGSA-0353 | F | UI |  | C | U |  | None |
| 05/22/05 | 17 | 2 | HGSA-0354 | M | UI |  | C | U | L | None |
| 05/22/05 | 17 | 2 | HGSA-0355 | M | UI |  | C | U |  | None |
| 05/22/05 | 17 | 2 | HGSA-0356 | M | UI |  | C | U |  | None |
| 05/22/05 | 17 | 2 | HGSA-0357 | U | UI |  | C | U | R-PD | None |
| 05/22/05 | 17 | 2 | HGSA-0358 | F | NT | TSST | S | TC |  | None |
| 05/22/05 | 17 | 2 | HGSA-0360 | F | UI |  | C | U |  | None |
| 05/22/05 | 17 | 2 | HGSA-0361 | M | UI |  | C | U | R-PD | None |
| 05/22/05 | 17 | 2 | HGSA-0362 | F | TR | ?S?? | T | TR |  |  |

Legend:

Research events are 1-2 days in length; thus, Day Number specifies which day it was within a research event.

For Sex, M=male, F=female, and U=unknown.

The Sighting Type can be UI=initial sighting of an untagged animal, UR=return sighting of an untagged animal (*can only be on day 2 of the same event), NT=newly tagged shark, and TR=returning tagged shark.

Tag Shape Sequence is the unique sequence of shapes attached to the visual marker tag. For example, CSCS is circle-square-circle-square. The first 5 tags had no shapes and are denoted as SP-n-ll where n=a unique number and ll=tag location.

ID Confidence specifies how the shark was identified for each research date. S=identified by tag shape sequence, C=confidently identified by 3 or more markings, T=tentatively identified by 1-2 markings or the observation was uncertain.

Equivalency details whether or not a tagged shark would have been identifiable if it had been untagged. U=untagged shark, TC=tagged shark which could be confidently identified by markings alone, TT=tagged shark which could be tentatively identified by markings, TU=tagged shark which was otherwise unidentifiable, and TR=returning tagged shark.

Orphaned Side – Potential Duplicate denotes whether or not the sighting was an orphaned side, which side of an orphaned side was seen completely, and whether it was a potential duplicate (PD) of other orphaned side sharks.

$ Additional data was used to determine this shark was not a potential duplicate. For example, HGSA-0264 is female and HGSA-0037 is male so they are unique.
